# Child Labor and Psychosocial Wellbeing: Findings from Ethiopia

**DOI:** 10.3390/ijerph19137938

**Published:** 2022-06-28

**Authors:** Cécile Fanton d’Andon, Claire Greene, Catherine Pellenq, Tesfahun Melese Yilma, Muriel Champy, Mark Canavera, Chiara Pasquini

**Affiliations:** 1 Care and Protection of Children Learning Network, Program on Forced Migration and Health, Heilbrunn Department of Population and Family Health, Columbia University Mailman School of Public Health, 60 Haven Avenue, B2-221, New York, NY 10032, USA; mc3718@cumc.columbia.edu (M.C.); chiarapasquini1@gmail.com (C.P.); 2 Program on Forced Migration and Health, Heilbrunn Department of Population and Family Health, Columbia University Mailman School of Public Health, 60 Haven Avenue, B2-221, New York, NY 10032, USA; mg4069@cumc.columbia.edu; 3 Laboratoire de Recherche sur les Apprentissages en Contexte/Research Laboratory on Learnings in Context, Grenoble-Alpes University, LARAC/1251 Avenue Centrale, Domaine Universitaire, 38000 Grenoble, France; catherine.pellenq@univ-grenoble-alpes.fr; 4 Dabat Research Center, Department of Health Informatics, Institute of Public Health, University of Gondar, Gondar 6200, Ethiopia; tesfahun.melese@uog.edu.et; 5 Department of Anthropology, Aix-Marseille University, 13015 Marseille, France; muriel.champy@univ-amu.fr

**Keywords:** child labor, child wellbeing, psychometrics

## Abstract

For children who work, there has been little research into the intricate relationship between their home lives and their work lives and the implications that this relationship might hold for their psychosocial development and functioning. This cross-sectional study was conducted in the Amhara region, Ethiopia, between March and April 2020 on a sample of 1311 working children with the aim, in part, of exploring ways in which various dimensions of children’s psychological wellbeing are influenced by their working conditions and their family contexts. In addition to collecting data on some personal traits, family relationships, home environments, and detailed occupational characteristics, we gathered information on psychosocial wellbeing using 22 items from the Instrument for the Psychosocial Assessment of Working Children (IPAC). Exploratory factor analysis enabled us to identify five factors characterizing the dimensions of psychosocial wellbeing: work-related self-esteem, work-related stress, workplace supervision, emotional and somatic wellbeing, and self-determination. Linear regressions of these factors were then conducted on social, occupational, and environmental variables. We found that all dimensions of psychosocial wellbeing were significantly associated with the children’s working conditions. Of particular interest, work-related dimensions of wellbeing, such as stress, self-esteem, and supervision, were significantly associated with the characteristics of the home and family environment. These findings illustrate that work and working conditions must be considered jointly, along with family life and home environments, as factors in both environments affect working children’s socioemotional development and wellbeing. They also strengthen the call for a systemic approach to protecting children involved in child labor, in which families are central to all discussions.

## 1. Introduction

Despite the ratification of the International Labor Organization’s Convention 182 on the worst forms of child labor [1] by the Ethiopian Government in 2003, occurrences of hazardous and illegal forms of child labor remain high in the country. According to a 2015 National Child Labour Survey, 51% of children in Ethiopia were working. In the Amhara region, this figure rises to 64%, with 30% of children involved in hazardous child labor [2]. While these figures represent a large share of children at risk of physical and mental harm as well as injuries related to child labor, they are also likely to represent underestimations. Being by nature illegal and often hidden, the worst forms of child labor tend to be systematically underestimated in studies measuring magnitude. Additionally, some of the risks associated with more visible forms of work receive less attention, as is the case of psychosocial risks associated with child labor [3].

Articles 3 and 4 of the Convention define the worst forms of child labor as practices similar to slavery or the use of children for illegal activities, along with all practices likely to “harm the health, safety, or morals of children”. Signatory countries are responsible for identifying what these worst forms of child labor encompass in their national contexts, opening a large field of research on how different work forms affect child health and wellbeing. This paper presents findings on the psychosocial wellbeing of children involved in child labor in Ethiopia’s Amhara region, with the objective of advancing knowledge around risks and protective factors affecting these children’s wellbeing to support the design of effective policies better tailored to their unique needs.

This article studies various dimensions of working children’s self-reports of wellbeing and examines how these dimensions are related to their working conditions and family situations. Specifically, this study aims to: (1) identify dimensions of psychosocial wellbeing among adolescent children involved in child labor in Ethiopia, and (2) explore how individual-, family-, and occupational-level characteristics are associated with these various dimensions of working children’s psychosocial wellbeing.

### 1.1. Literature Review

The below section seeks to understand how the study of child laborers’ wellbeing fits into the larger body of research focusing on children’s wellbeing and reveals the need for rigorous quantitative studies exploring the link between child laborers’ wellbeing and their work and sociodemographic characteristics in the Global South.

#### 1.1.1. Study of Child Wellbeing

Child protection as a research field underwent major shifts at the beginning of the 21st century as the emphasis moved from survival and long-term perspectives, or well-becoming, to focusing on the current state and quality of life, or wellbeing [4]. While theoretical and political controversies around the concept and measurement of wellbeing still exist, the field has progressively acknowledged that the experience of the child is key in understanding wellbeing; there is also growing consensus that indicators collected from adults cannot be the sole source of information concerning child wellbeing [5,6,7]. As children are actors in society, researchers have advocated for their participation in research, developing methods for eliciting subjective indicators of wellbeing. This evolution is a consequence of increasing recognition of and attention to child participation as a foundational right [8,9,10]. Large surveys have studied children’s wellbeing, including Children’s Worlds’ International Survey of Children’s Wellbeing, conducted three times over 35 countries since 2011 [11], and the World Health Organization’s “Health and Behavior in School-Aged Children” study, which has taken place in countries throughout Europe and in Canada every four years since 1984, with the last round covering 50 countries [12]. Findings from these surveys point to cultural differences in children’s perceptions of their wellbeing. They also suggest that wellbeing is influenced by poverty, inequality, quality of family and social relationships, gender, and age, with girls and teenagers being more at risk of demonstrating relatively poor wellbeing [13].

#### 1.1.2. Study of Working Children’s Wellbeing

The study of the impact of work on child wellbeing has greatly benefited from the research of Woodhead, who argues for the need to understand the relationship between work and wellbeing within the broader context of children’s lives [14]. Woodhead defines five broad domains of psychosocial wellbeing: cognitive abilities, personal security, personal identity, sense of personal agency, and emotional and somatic expressions of wellbeing. He suggests that children’s work can impact these dimensions of psychosocial wellbeing positively if it provides a stable, predictable environment where a child learns skills that are valued in their society. Inappropriate forms of work, excessive expectations from families and employers, or lack of protective factors can, on the other hand, be a source of disruption to the child’s emotional stability.

Threats to the wellbeing of working children in developing countries such as Ethiopia, which relies heavily on its workforce for intensive physical work, are a major public health concern but have rarely been explored through large-scale quantitative studies [15]. The cross-sectional design of most studies limits the causal interpretation of their findings [16]. Among the few studies that have been conducted, emotional disorders are found to be more common among child laborers than non-laborers [17,18,19,20]. Time spent on hard and repetitive work, over which children have little control, is identified as a cause of demoralization [21] and exhaustion [22]. Work that keeps children away from their homes or peers is a cause of isolation [14,21,23], and work that forces children to leave school causes the loss of social and educational opportunities [14,21,22]. Work can also be a place where the child is subjected to abuse and violence [24,25] and increased risks of accidents [26,27,28].

Despite acknowledgment that the psychosocial wellbeing of working children is affected by their working conditions [14], few quantitative studies have explored the effect of sociodemographic characteristics on child laborers’ mental health. The association between work and poor mental health has been found to be weaker past the age of 13 [29]. Being a girl has been found to have an overall negative effect with respect to all aspects of wellbeing among working children [20,30]. Combining work and school was found to be associated with poorer mental health outcomes in a study of Jordanian child workers [31], while attending school was found to have a potential protective effect on children working in the brick industry in a multi-country study [20]. One recent analysis performed in India suggests a strong negative impact of work as a whole on child wellbeing, with work being associated with decreased emotional wellbeing and decreased happiness [32].

## 2. Materials and Methods

### 2.1. Design and Setting

This research study analyses data collected as part of an impact evaluation of a project targeting child workers that was implemented by World Vision Ethiopia in the Amhara region. The Amhara region has the second-highest percentage of children involved in hazardous child labor, as per the 2015 National Child Labor Survey [2]. A community-based cross-sectional quantitative survey, complemented by qualitative data collection, was conducted in three districts of the Amhara region from March until April 2020 to provide baseline measures for the impact evaluation. This study was conducted with a selected sample of children involved in or at risk of being involved in the worst forms of child labor. A sample of 3229 children, together with their heads of household, were selected for the study. The main criteria for selection into the study were economic vulnerability associated with the engagement of children in hazardous work or illegal forms of work, and at least 50% of study participants had to be female. The study participants were identified by local child protection units and registered by World Vision Ethiopia.

#### Ethics

The study was conducted according to the guidelines of the Declaration of Helsinki and approved by the Institutional Review Board of Columbia University (IRB-AAAS8342, 02/20/2020) and by the Ethiopian Society of Sociologists, Social Workers, and Anthropologists (IRB/RSSSWA/002/2020). A team of experienced enumerators was trained for 10 days. Their training modules covered requesting consent, the survey protocol, child labor basics, interview techniques with children, child safeguarding, and reporting mechanisms. Children who were 13 years old or older participated in the survey directly. To gather information on children below 13 years of age, the survey interviewed their primary caregivers. Adult and child respondents provided consent for themselves, and for child respondents, we secured the consent of the children’s caregivers as well.

### 2.2. Measures

All data analyzed in this study were self-reported with two quantitative questionnaires. The first questionnaire involved a series of modules aimed at capturing household and household head characteristics, and the second captured the children’s working conditions and wellbeing. Modules were translated into Amharic and translated back into English to verify translation accuracy. The two questionnaires were then pre-tested on a subsample of 20 respondents and the content adjusted accordingly. The questionnaires were programmed using the surveyCTO platform, with skip patterns and logic rules to limit potential data entry errors and administered via electronic tablet by enumerators from the area of implementation. Children’s literacy did not impact their ability to complete the survey as the enumerators were responsible for completing the questionnaire based on the children’s answers.

The heads of the children’s households were systematically asked the household module questions. For ethical reasons, only children aged at least 13 years old were interviewed directly, so the module on children’s characteristics was sometimes completed by their primary caregiver. This study only includes data collected from children above the age of 13 and their primary caregivers, with a sample size of 1311 children. For this analysis, the enumerators captured detailed information only about the child’s main work activity. The child’s main work activity was defined as the paid activity on which the child was spending the most time or, if the child did not have a paid activity, the activity on which the child was spending the most time.

To complement the quantitative analysis, in-depth interviews were run with a subsample of 25 children and 20 caregivers. This subsample was recruited based on both objective criteria, such as age and type of occupation of the child, and interviewers’ subjective opinions, following quantitative data collection, on child or caregiver willingness and ability to share information about their lives. The interview guides developed for that work cover the child and family history, work, and education. Transcripts were translated into English and coded thematically by one researcher. Qualitative findings were used to complement the quantitative analysis in the current paper.

#### Measure of Child Laborer Wellbeing

The development of tools dedicated to measuring child laborers’ wellbeing has been promoted by the International Labor Organization (ILO) and has led to the establishment of the Instrument for the Psychosocial Assessment of Working Children (IPAC). The IPAC was built on extensive research by Leka and Jain in reviewing and selecting the tools used to assess psychosocial risk; they suggested that the tools used for adults cannot be transposed for use with children. It also uses the SAFE framework developed by Betancourt [33] and the “Child and Youth Resilience Measure” (CYRM-28) [34]. The IPAC was created by a group of experts whose objective was to develop a tool appropriate for children between 11 and 18 years old that could be used cross-culturally and employed by non-specialists. The tool was tested on different samples and was validated in Pakistan and Nepal in 2015 [3,20].

This study uses a subsample of the IPAC to capture wellbeing information. For this exploratory work, 22 Likert scale items were selected to limit the questionnaire length. They were selected with the aim of reducing redundancy with other parts of the questionnaire, matching the study’s overall research priorities, and taking into account feedback from the research team regarding the children’s understanding of the items. For each item, children expressed to what extent a given sentence applied to them. They could select Always, Often, Sometimes, or Never. The rating scale of the Likert scale was distributed from 1 (=never) to 4 (=always). The distribution is reported in Appendix A. For the analysis, all items were coded so that in the data, 1 would be the worst outcome and 4 the best outcome relative to wellbeing; therefore, negative items were reverse-coded.

### 2.3. Statistical Analysis

Item variance, skewness, and inter-item correlation were examined prior to conducting exploratory factor analysis. With a sample of 1311 individuals and 22 items, the sample size was adequate for exploratory factor analysis. Preliminary principal component analysis enabled us to select a five-factor model. Both Schwarz’s BIC and Akaike’s AIC statistics have their smallest values for the five-factor model, suggesting that five underlying factors best explain the variance and that this five-factor model achieves the best balance between goodness of fit and parsimony. Factor coefficients were predicted using exploratory factor analysis: we applied factor analysis to determine which items best fit each of the factors, allowing us to understand what dimension of wellbeing a given factor embodies. Exploratory factor analysis was followed by a non-orthogonal rotation to allow for better interpretability of the model while still enabling factors to be correlated. The analysis, therefore, associates a level of the underlying constructs for each individual, attributing a factor score for each identified factor.

### 2.4. Model

To examine correlates of psychosocial wellbeing, we selected covariates from three categories: personal information of children, family circumstances, and working conditions. The objective of this analysis is to understand the magnitude of the linear relationship between various dimensions of children’s wellbeing and specific aspects of children’s family, personal, and work correlates.

The model we are testing, therefore, is a simple linear relationship linking the psychosocial wellbeing dimension ($y_{i}$ below) with individual covariates $\left(x_{i}\right)$. Wellbeing dimensions are latent factor scores. Individual covariates are comprised of personal characteristics ($x_{pi}$), family circumstances ($x_{fi}$) and work characteristics ($x_{wi}$); β is our measure of interest as it characterizes the linear relationship between covariates and latent factors scores.

(1) $$\mathrm{Linear}\;\mathrm{regression}\;\mathrm{model}\;y_{i}=\alpha +\beta x_{i}+\varepsilon$$

(2) $$\mathrm{Covariates}\;x_{i}=x_{pi}+x_{fi}+x_{wi}$$

### 2.5. Covariates

The individual-level covariates included in the analysis are gender, age, current schooling status, and occurrences of any sicknesses in the past year.

Family and community circumstances include a wealth index, an indicator capturing whether the child lives with their parents, an index of perceived family support, a perceived friend quality index, and an indicator of social norms around child labor in the community.

To study work characteristics, we included information about socialization at work (whether a child was working with others or alone), information about income from work, and information about risks associated with work. Since many children reported attacks occurring on the way to work, we included whether the child had to travel more than 30 min to work. To compute risk in the workplace, the study captured occurrences of violence and a more formal indicator of hazards based on the ILO definition of hazardous work, slightly tailored for specific risks incurred by children involved in *khat* collection.

Five indexes were created for the study: a wealth index, a family support index, a friend quality index, a hazardous work index, and a violence index. Each index was created by averaging individual items that contributed to it (see Table 1 for details of items used to build each index). They were then standardized using the following formula, where *µ* is the mean and σ the standard deviation:

$$x_{z}=\frac{x-\mu }{\sigma }\;$$

## 3. Results

### 3.1. Covariates

The 1311 children who took part in the survey were, on average, 14.5 years old at the time of the survey. Forty-seven percent (47%) of the respondents were girls. On average, 79% of the children were enrolled at school, with proportionally more girls than boys enrolled.

Analysis of family characteristics indicates that boys in our sample have slightly more supportive relationships with their families than their female counterparts, with a higher score on the family support index and with 53% of girls reporting fighting with household members as opposed to 48% of boys.

Overall, boys seem to work in harder conditions with a longer average commute than girls, with the highest scores on violence and hazards faced at work, with differences being significant. As many children reported working for free, the average monthly income was very low (0.18USD), with boys earning significantly more than girls.

**Table 1:** Characteristics of the sample.

	Girls	Boys	All	Difference between Girls and Boys
**Individual characteristics**				
Age	14.47	14.58	14.53	
Currently enrolled at school (yes = 1)	84%	74%	79%	Significant
Was sick in last 12 months (yes = 1)	60%	65%	63%	Significant
**Family and community characteristics**				
Wealth index (standardized) ^1^	−0.05	0.04	0.00	
Family support index (standardized) ^2^	−0.05	0.04	0.00	
Child has fights with his household members	53%	48%	51%	Significant
The child does not live with at least one of his parents (yes = 1)	61%	57%	59%	
Child’s community views working children favorably (completely agree = 1) ^3^	49%	49%	49%	
Friend quality index ^4^	−0.01	0.01	0.00	
**Work characteristics**				
Child is working alone (yes = 1)	37%	40%	39%	
Child’s monthly income (in USD)	0.15	0.20	0.18	Significant
Child walks more than 30 m per day to go to work (yes = 1)	24%	38%	31%	Significant
Child has food or water at work (yes = 1)	71%	62%	66%	Significant
Child is engaged in hazardous work (index standardized) ^5^	−0.10	0.08	0.00	Significant
Child is a victim of violence at work: Violence index (standardized) ^6^	−0.04	0.03	0.00	
Child’s main activity is casual work (yes = 1) ^7^	7%	20%	14%	Significant
Child is engaged in khat collection (yes = 1)	9%	12%	11%	Significant
Child is engaged in domestic work for their household (yes = 1)	9%	1%	5%	Significant
	611	700	1311	
*Reading: in our sample, 515 girls were enrolled at school, which represents 84% of the girls in the sample.*				

^1^ The Wealth index is built on items indicating household possessions and housing quality based on DHS items used in rural Ethiopia. ^2^ Built on four indicators: family advises or encourages the child, child has fights with family, family talks with child about how things are going at school, family leaves the children with time to play. Index is standardized for ease of interpretation, mean = 0, SD = 1. ^3^ Data collected from child’s caregiver. ^4^ Built on four indicators: child has friends they can talk to about important things; child has friends they can rely on for emotional support; most of my friends are working; most of my friends consume khat (recoded). Index is standardized for ease of interpretation. ^5^ Built on a series of indicators characterizing hazards: very noisy workplace, bad lighting (cannot see properly or light too bright), working with dangerous or poisonous substances, working with dangerous machinery or tools, working with or near dangerous animals, working underground or at heights, working in a very cold or very hot place, fear that a person may hurt you, not allowed to go home or leave the workplace even when you feel bad, carrying heavy loads, cutting khat leaves with your teeth, feeling dizzy or excited because of the smell of substances at work. Index is standardized for ease of interpretation. ^6^ Built on indicators of violence at work: child is shouted at, insulted, beaten or physically hurt, or harassed while at work. Index is standardized for ease of interpretation. ^7^ Khat is a plant that is chewed by consumers as a stimulant and is said to have addictive properties. Children work in plantations to collect the leaves. As they collect it, they often cut the leaves with their mouth, potentially putting them at risk of developing addictions.

### 3.2. Measurement of Psychosocial Wellbeing

The factor structure obtained with exploratory factor analysis identified five factors. The highest loading items allow us to understand the underlying construct for each factor. Factor loadings are reported in Table A2 in Appendix B and enabled us to characterize the underlying constructs represented by the factors. Three factors characterized work-related wellbeing: work-related self-esteem, work-related stress, and supervision. The two other factors captured the emotional and somatic expressions of wellbeing and feeling of agency. This result aligned to a certain extent with the results of Pellenq and colleagues (2021) [20], also based on the IPAC but from data collected in southeast Asia.

The oblique rotation enabled the factors to be correlated; the correlation matrix is reported in Appendix B in Table A3. The highest correlations were found between the emotionality factor and the stress factor and between self-agency and supervision.

#### 3.2.1. Factor 1: Emotionality

This factor was built primarily from child-reported negative or uncomfortable emotional and somatic indicators, such as worrying, having trouble concentrating, sleeping, and feeling sad. We called this factor emotionality in reference to Pellenq and colleagues (2021), and it also relates to Woodhead’s concepts of “emotional and somatic expressions”. The highest loading items cover somatic symptoms such as body tension and dizziness as well as emotional symptoms such as worrying and feeling sad.

#### 3.2.2. Factor 2: Self-Agency

This composite factor captures self-agency, such as feeling that one has high energy, time for oneself, and one is free to do what one wants. It relates to the concept of a sense of agency, as developed by Woodhead (2004).

#### 3.2.3. Factor 3: Work-Related Self-Esteem

This factor drew mainly on four items characterizing child self-competence and work-related social recognition. It relates to Woodhead’s concept of personal identity and valuation, along with those of cognitive abilities. The two highest loading items characterized the child’s work-related pride and whether the child enjoys the work.

#### 3.2.4. Factor 4: Work-Related Stress

This factor drew mainly on three items characterizing a feeling of pressure and tiredness. We named it “stress” in reference to Pellenq and colleagues (2021) [20], who identified a similar factor.

#### 3.2.5. Factor 5: Supervision at Work

Supervision at work mainly drew on two items characterizing the children’s feelings of oversight, guidance, and, ultimately, safety. This concept relates closely to Woodhead’s concept of personal security through positive adult/children relationships. It relies on two items: whether children feel supervised and whether they feel people are teaching them how to work.

Having identified five factors: emotionality, agency, work-related self-esteem, work-related stress, and supervision, we then analyzed the magnitude and type of linear association between personal, family, and work characteristics and the psychosocial wellbeing of children.

### 3.3. Association between Child, Family, Community, and Work Characteristics with Child Workers Psychosocial Wellbeing: Linear Regression Analysis

Table 2 below provides the results of linear regression of the wellbeing factors on some of the personal, family and community, and work-related correlates selected above. We conducted a linear regression based on a t distribution.

#### Findings by Dimension of Psychosocial Wellbeing

i. Factor 1: Emotionality

This factor includes sadness, anxiety, and anger but also physical signs of negative emotions, such as tension, loss of appetite, and difficulty sleeping. The R-squared score suggests that the model explains 34% of the variance of the factor. Results of the linear regressions by categories of covariates for that factor are reported in Appendix C in Table A4. In line with the literature, girls are found to be more at risk of developing negative emotional signs (β = −0.18). Our findings suggest that getting older is associated with a small but statistically significant decrease in wellbeing, which might indicate that children have to cope with higher workloads as they grow up and might become demotivated by the absent view of a better future. One aspect of children’s personal characteristics that correlates strongly with emotionality is the occurrence of sicknesses in the past twelve months (β = −0.33). This result suggests that children’s physical and emotional health are linked; it may also illustrate anxiety related to illness and potential loss of income. Indeed, sick children are at risk of losing their source of income, as illustrated by the quote of an employer below.

“If a child isn’t capable of doing his activities properly within the working hours we agreed upon, due to… different reasons, the only thing that we can do is to calculate his rate and fire him or her.”

[Employer 35, Gondar Zuria]

Data show that not living with at least one parent and having fights with their family members is significantly associated with decreased emotional stability (β = −0.17 and β = −0.13, respectively), illustrating the destabilizing effects that tensions and separation from family can have on children’s wellbeing.

Our results support the idea that working children’s wellbeing is greatly correlated with their working conditions and that children suffer from risks associated with work. The emotionality factor shows strong and statistically significant associations with two dimensions of a child’s work that characterize risk: being engaged in hazardous work (β = −0.23) and being a victim of violence in the workplace (β = −0.21). Being engaged in hazardous work and being a victim of violence at work appear to deprive children of control in their work environments and seem to generate stress and anxiety. In interviews, many children denounced the difficulties they faced at work, as illustrated by the quote below from a 14-year-old girl engaged in street vending and a boy, 17, engaged in cattle keeping.

“Children should not work in activities that are beyond their physical and mental capacity.”

[Girl, 14, Libo Kemkem]

“In the future, I don’t want my children to suffer or engage in the work I am doing now.”

[Boy, 17, Gondar Zuria]

Being engaged in casual work is also strongly and negatively associated with the factor of emotionality. In such forms of work, children engage in what is available to them on a daily basis; they usually do not even know their employers. Children are not always sure they will find work and endure the risk of spending the day waiting for someone to employ them and returning home without any earnings. The lack of secure relationships and consistency associated with that way of working seems to generate anxiety (β = −0.18).

In the area where the survey took place, caregivers suggested that the availability of food and water was an important protective factor in the workplace. We included this indicator in our model and found that being provided with either food or water or both seems to correlate positively with children’s emotionality (β = 0.11), which indicates that workplaces that provide food and water are places where children feel more secure.

ii. Self-agency

In this dimension, our results indicate that children thrive when they are agents of decisions and might suffer in situations where they have little control, where the cost of working exceeds monetary gains, and where they do not feel they can change their situations. The R-square scores suggest that the model explains 18% of the variance of the factor. Results of the linear regressions by categories of covariates for that factor are reported in Appendix C in Table A5. Being enrolled at school is negatively associated with children’s feelings of self-agency (β = −0.13). As illustrated by the quote below, having to attend school and work does not leave children time for homework and even less time for rest.

“When I’m in class I usually think of what I’ll face after school. The amount of food that my employers will give me. The difficulty of the task and the insufficient spare time for study. […] When I go to school, I am present physically, but all my attention is on my work. When I leave school, I come back home running because I feel like my employers might punish me for being late.”

[Girl, 15, Gondar Zuria]

Interestingly, having fights with other household members and not living with at least one parent both have a moderate but highly significant positive association with self-agency, suggesting that disagreement with household members might either create or, conversely, result from a greater sense of autonomy in children (β = 0.16). However, having a supportive relationship with the family has a much larger and positive association with children’s self-agency (β = 0.23). Friend support is also associated with an increase in self-agency (β = 0.05).

The statistically significant positive association between the self-agency factor and community norms supporting child labor (β = 0.13) illustrates an interesting point. In the areas of Ethiopia where the study took place, entrusting children with work is perceived primarily as “good parenting”. In interviews, caregivers insisted on the importance of children developing a “culture of work”, thus establishing work as a key aspect of children’s socialization. Children perceive their participation as legitimate and an opportunity to learn skills, as illustrated below by quotes from working children.

“Work is one of the best ways to learn about the world.”

[Girl, 15, Gonder Zurya]

“Children should adopt a working culture. This will help them to be a strong and hard-working person in their adulthood. A child who isn’t working and supporting his family during his childhood will be a lazy person who is expecting everything from other people.”

[Girl, 14, Libo Kemkem]

While children, to a certain extent, find it empowering to work and learn new skills, they might lose their sense of agency when having to work in conditions that they do not decide on for themselves. Having to commute more than 30 min per day (β = −0.14) and working alone (β = −0.12) are significantly associated with a lower sense of self-agency. Working alone is negatively associated with self-agency. Working alone tends not to be the children’s main choice as they prefer working with peers or other family members. In the interviews, the children valued the social integration that some forms of work provide, such as sharing household chores with family members or fetching water with friends. A child working alone might feel excluded and lack personal growth, as illustrated below by a child working in cattle keeping, an activity that entails long, lonely hours in the countryside.

“What skills can I learn from this tiresome work? It is a cattle herding. I did the same work when I was at my parents’ home. There is nothing new.”

[Boy, 13, Dera]

Being engaged in hazardous work (β = −0.05) and violence in the workplace (β = −0.06) both displayed a small but statistically significant and negative association with psychosocial wellbeing. The lack of predictability of casual work can explain the significant negative association this covariate has with self-agency (−0.21). Receiving payment for work has a slightly significant positive association with children’s self-agency (β = 0.16). Children reported that the payments enabled them to decide on what to provide for themselves and their families and provided a sense of fairness in the situation.

iii. Work-related self-esteem

This factor is built on feelings of appreciation and pride regarding work. The R-square scores suggest the model explains 18% of the variance of the factor. Results of the linear regressions by categories of covariates for that factor are reported in Appendix C in Table A6. Working children acquire positive self-image and self-esteem when their activity is valued and when they feel competent in what they are doing. The positive association reported with family support (β = 0.14) is, therefore, expected. A community’s positive views of children’s work are also positively and significantly associated with that dimension of wellbeing (β = 0.10). This result is reflective of caregivers and community members who value children’s work and also enable children to make sense of their role as workers contributing to their community. Being a girl is associated with lower work-related self-esteem, which might illustrate that girls’ work may go less noticed or may be less valued than work performed by boys. We would expect being paid to be perceived as a reward and recognition of work performed, but our model did not identify a significant association.

When there is no one around to encourage or value the work done by the child, or when the child is a victim of abuse in the workplace, the child’s engagement in work might be a threat to their self-esteem. This result is illustrated by the significant and negative association between self-esteem and working alone (β = −0.13), commuting (β = −0.27), and being subjected to abuse (β = −0.14).

In addition to working conditions, the type of work activity might affect children’s self-image as well. Being engaged in domestic work for one’s household is weakly but positively correlated with children’s self-esteem (β = 0.22). This result may emerge not only because domestic work consists primarily of tasks that correspond to children’s skillsets but also because these are associated with positive community norms of care, as illustrated by the quote of a girl from the study area.

“I am pleased to support my grandparents. They are retired, and they don’t have children who live with them, so they need my support. It is my duty to serve them with all my capacity.”

[Girl, 15, Gondar Zuria]

Khat collection, on the other hand, is widely perceived as a “bad” form of work by the community. Therefore, engagement in khat collection might constitute a threat to a child’s self-esteem (β = −0.2). This result is illustrated by the quote of a caregiver below, whose son works in khat collection and who disapproves of this activity.

“His friends made him drop out of school and start the work. We don’t want him to work. He doesn’t even support us! He just gambles with his bad friends.”

[Father, 66, Gondar Zuria]

iv. Work-related stress

The work-related stress factor is mainly built on items related to being asked to perform unreasonable amounts of work by capturing the feelings of being under pressure, being tired, and boredom at work. The R-squared score suggests that the model explains 35% of the variance. Results of the linear regressions by categories of covariates for that factor are reported in Appendix C in Table A7. The small negative coefficient (β = −0.03) on age suggests that children have to cope with a higher workload as they grow up. Girls seem to report enduring higher stress levels at work (β = −0.14), a result that suggests that they are being asked to perform more than their male counterparts, particularly in taking care of household members or various household chores, as illustrated by this quote from a boy of the study area.

“The work burden in this area is much higher on girls than boys. The boys are not mostly expected to work in the house but the female is expected to work and help her household in household chores. This burden of work on females sometimes forces them to miss their school, which leaves the girls stressed, and they even become angry and feel helpless.”

[Boy, 16, Gondar Zuria]

Occurrences of fights with household members (β = −0.09) are negatively associated with work-related stress, suggesting a link between children’s level of stress and the quality of their interactions with their close family circle. Community norms valuing children’s work are negatively associated with work-related stress, suggesting that these community norms might result in pressure on children to engage in forms of work that are not adapted to their strength and age. The work stressors mentioned above, hazards (β = −0.17) and violence (β = −0.26), are associated with higher child-stress levels. Commuting is also a work hazard, with many children interviewed reporting fear of attacks on the way from or to work, pain associated with walking for long hours in high temperatures, or accidents happening while commuting at night. These dangers are reportedly higher in urban environments and tend to explain the negative association between work-related stress and commute times (β = −0.19). Interestingly, earning an income is associated with an increased level of stress (β = −0.15), suggesting that children who earn an income are subjected to more pressure to work efficiently than their non-paid counterparts.

v. Supervision

The supervision factor is mainly built on items capturing whether the child feels supervised and is taught how to perform the work well. The R-squared score suggests that the model explains 16% of the variance. Results of the linear regressions by categories of covariates for that factor are reported in Appendix C in Table A8. Being raised in a supportive household where adults take time to talk with the child and are concerned about their working conditions is strongly associated with a higher level of feeling of supervision at work (β = 0.26), suggesting supportive households might pay more attention to a child’s working conditions. Interestingly, children who reported fighting with their household members also reported higher levels of supervision at work (β = 0.13). Additionally, in line with intuition, working alone (−0.18) and casual relationships with employers (−0.24) are associated with a decreased sense of supervision. Work involving long commutes is also associated with a decreased sense of supervision (β = −011), which might relate to the fact that children are less supervised when they work outside their community in places where they do not know anyone.

## 4. Discussion and Limitations

### 4.1. Discussion

In this study, we examined the construct validity of a subset of the IPAC among children in Ethiopia. In our sample, we identified five dimensions of child psychosocial wellbeing. We identified three that are specific to work (self-esteem, stress, and supervision) and two others that are not specific to work (emotionality, self-agency). Because we used only a subset of items, it was expected that we would not capture all factors that have been found in the research of others, such as Pellenq and colleagues (2021) [20]; however, we identified four of the six they identified, an outcome that increases confidence in the reliability of the tool.

With a sample of 1311 children from the same area and highly detailed information on the children’s working conditions, this analysis studied the main correlates of social–emotional wellbeing for working children as reported by them. It sought to advance the knowledge on how children’s personal, family, or work characteristics might affect their wellbeing in a granular way.

These results tend to confirm a negative association between child workers’ wellbeing and hazardous conditions, as identified by the ILO and adapted to the Ethiopian context, and violence in the workplace. Of the work characteristics included in the model, two features of work that have, to date, received little attention in the literature stand out as being negatively associated with most dimensions of wellbeing. The first of these is commute time, and the second is engagement in casual work. Our results suggest that children who have to walk long distances and those looking for work on a daily basis without any ability to select their employers incur additional risks to their safety and wellbeing.

Our results also illustrate the difficulty of categorizing the worst forms of child labor through the sole prism of hazards known to be associated with the activities studied. Children might suffer from an activity or, conversely, enjoy it, depending on a number of features: their age, strength, the distance to be traveled to reach the workplace, the possibility of learning new skills, the possibility of sharing the activity with peers, the time spent working every day, the relationship with their employer, and the recognition they get from engaging in such work. The ways in which working conditions will impact children’s different dimensions of wellbeing can, therefore, only be understood when embedded in their wider personal and family contexts. Our findings suggest that families, unsurprisingly, can serve as a key protective unit for children’s wellbeing by potentially offering a predictable, encouraging, and caring space in which children can feel supervised and develop emotional balance, self-esteem, agency, and coping mechanisms for stress. Our results show that supportive relationships with adults in the family are a key factor in enhancing self-esteem, empowerment, and feelings of supervision among children who work. They also indicate that growing up in a community that favors a “culture of work” for children enables working children to develop a positive self-image. Girls are found to be more at risk of developing negative feelings, poorer work-related self-esteem, and more work-related stress.

These findings have implications for policies trying to address child labor and improve children’s wellbeing. First, they might call for a “risk reduction” approach to supporting working children. While interventions enabling children to be freed quickly from the most harmful forms of work are necessary, more focus should be put on the development of programs aimed at reducing identified risks at work and on the way to work, alleviating stress factors, and enabling children to contribute to their families in a way that is safer and more fulfilling. Our results suggest that parenting or caregiving interventions that equip parents with the knowledge and tools to better protect their children from harm are relevant. In complex and changing contexts, however, children might live with people other than their parents. Thus, such interventions need to address the community at large to reinforce existing child protection mechanisms. Programs targeting normative change may focus on specific risks that are relevant to certain contexts rather than condemning children’s work as a whole. Indeed, condemning children’s work could change children’s self-perception from being actors to victims, and, in the absence of effective support for children to transition out of work, such a change in self-perception might decrease their self-esteem. Our findings seem to reiterate the need for programs using a gender lens and focusing on isolated children and children not living with their parents. Finally, these findings call for a child-centered approach in response to child labor. Indeed children, similar to all social actors, have their own understanding of their social contexts and make sense of them through their interactions with others, work representing part of these interactions. Therefore, approaches in response to child labor should consider children’s voices to inform the development of policies that will effectively match the reality of their needs and improve their wellbeing.

### 4.2. Limitations and Further Research

The program through which these data were collected and analyzed aimed to support caregivers and children jointly and did not allow for the registration of completely marginalized children, thus excluding from the study vulnerable children living on the streets or working in hidden or bonded labor. Additionally, the questions were put to children at least 13 years old and, hence, did not inform us of the wellbeing of younger children. The study results are thus valid for children known and identified by their communities as vulnerable and at least 13 years old. The results might not be generalized to other age groups. Further studies could examine how different covariates relate to children’s wellbeing at different stages of development.

By using a reduced version of the IPAC on a large variety of children’s occupations, the present study can neither contribute to the further validation of the tool nor explore all dimensions of children’s wellbeing, as measured by the 47 items of the IPAC. It nevertheless suggests that the IPAC can be used with child workers involved in different types of work. Further studies could explore how to use all 47 items and deepen our understanding of how the tool could work in new contexts.

This article establishes relationships between children’s working and family conditions and wellbeing, but despite the large variety of correlates included and the relatively strong fit of the model, the analysis does not fully explain the various dimensions of wellbeing studied and can only suggest plausible causal relationships. Further studies could try to isolate the effect of work on children’s wellbeing by identifying exogenous sources of variation in either the propensity of children to engage in work or type of work performed by children. Random assignment to a program targeting child labor could also be of interest. Overall, longitudinal studies observing working children, as their situation evolves over time, are of great interest.

This analysis builds a unique model for the series of factors, while further analysis could identify best fits among different models for each factor. In addition, the analysis presents the potential effects of risks and protective factors as independent from each other. However, intuition suggests both that these factors are correlated and that cumulative risks will weigh more on children than each of the risks individually. Therefore, further research looking at the effect of combining multiple psychosocial and physical risks or the interaction between various risk and protective factors is needed to deepen our understanding of children’s wellbeing.

## 5. Conclusions

Our work shows that all dimensions of wellbeing are significantly affected by the working conditions of children, illustrating both the importance of work and working conditions in a child’s socioemotional development and highlighting key risk and protective factors. We also reveal that the dimensions of wellbeing linked to work, such as work-related self-esteem, supervision, and work-related stress, are significantly associated with the personal or familial characteristics of children. This understanding can pave the way for a systemic approach to protecting children involved in child labor by involving families in the discussions and taking a gendered approach to addressing the issue.

By uncovering some of the potential consequences of children’s personal, family, and work situations on wellbeing, the current study exposes a need for additional investigations on those dimensions of children’s psychosocial wellbeing in order to isolate the impact of work on children’s wellbeing and to understand the cumulative effects of risks on children’s wellbeing. Additional research on occurrences of violence in the workplace deserves more attention, and the accrued vulnerabilities of girls should also be studied and understood and then addressed at a programmatic level. While this research can only provide a snapshot of the situation and does not perform a long-term analysis of the risks associated with hazards to children’s wellbeing, further analysis could examine the long-term effects of exposure to risk and the protective factors identified in this study as children transition into adulthood. Further research should aim to capture children’s experiences in a granular way by ensuring that enough consideration is given to their voices during all phases of the research.

## Figures and Tables

**Table 2:** Association between child, family, community, and work characteristics and child workers’ psychosocial wellbeing: a linear regression analysis.

	Factor 1	Factor 2	Factor 3	Factor 4	Factor 5
	Emotionality	Self-Agency	Work-Related Self-Esteem	Work-Related Stress	Supervision at Work
**Child characteristics**					
Age	−0.04 ** (0.02)	0.02 (0.02)	0.02 (0.02)	−0.03 ** (0.01)	−0.02 (0.02)
Child is a girl	−0.18 *** (0.04)	−0.03 (0.05)	−0.14 *** (0.05)	−0.14 *** (0.04)	0.05 (0.05)
Child is enrolled at school	0.05 (0.05)	−0.13 ** (0.06)	−0.04 (0.06)	0.08 (0.05)	−0.1 * (0.006)
Child has been sick over the past twelve months	−0.33 *** (0.04)	−0.07 (0.04)	−0.05 (0.05)	−0.03 (0.04)	−0.04 (0.05)
**Family’s and community’s characteristics**					
Asset and wealth index	0.03 (0.02)	0.00 (0.02)	0.02 (0.02)	−0.02 (0.02)	0.04 (0.02)
Family support index	0.04 * (0.02)	0.23 *** (0.02)	0.14 *** (0.03)	0.04 ** (0.02)	0.26 *** (0.03)
Child has fights with household members	−0.13 *** (0.05)	0.16 *** (0.05)	−0.02 (0.05)	−0.09 ** (0.04)	0.13 ** (0.05)
Child does not live with at least one of their parents	−0.17 *** (0.04)	0.10 ** (0.05)	−0.06 (0.05)	−0.01 (0.04)	−0.08 * (0.05)
Child’s community views working children favorably	−0.03 (0.04)	0.13 *** (0.04)	0.10 ** (0.05)	−0.07 ** (0.04)	0.07 (0.05)
Friend quality index	0.02 (0.02)	0.05 ** (0.02)	0.01 (0.02)	0.02 (0.02)	0.01 (0.02)
**Work Characteristics**					
Child is working alone	−0.03 (0.04)	−0.12 ** (0.05)	−0.13 ** (0.05)	0.00 (0.04)	−0.18 *** (0.05)
Child’s monthly income (in USD)	−0.02 (0.08)	0.16 * (0.08)	0.13 (0.09)	−0.15 ** (0.07)	0.00 (0.09)
Child walks more than 30 min per day to work	−0.07 (0.05)	−0.14 *** (0.05)	−0.27 *** (0.06)	−0.19 *** (0.04)	−0.11 ** (0.05)
Child has food or water at work	0.11 ** (0.05)	0.06 (0.05)	0.12 ** (0.05)	0.10 ** (0.04)	0.10 ** (0.05)
Child is engaged in hazardous work (ILO index)	−0.23 *** (0.02)	−0.05 ** (0.03)	−0.01 (0.03)	−0.17 *** (0.02)	0.00 (0.03)
Child is a victim of violence at work (index)	−0.21 *** (0.02)	−0.06 *** (0.02)	−0.14 *** (0.03)	−0.26 *** (0.02)	−0.02 (0.02)
Child’s main activity is casual work	−0.18 *** (0.07)	−0.21 *** (0.07)	−0.23 *** (0.08)	−0.06 (0.06)	−0.24 *** (0.07)
Child’s main activity is *khat* collection	−0.01 (0.09)	0.05 (0.09)	−0.20 ** (0.01)	0.00 (0.08)	0.00 (0.09)
Child’s main activity is domestic work	−0.12 (0.01)	0.07 (0.11)	0.22 * (0.12)	0.08 (0.09)	0.06 (0.11)
Observations	1311	1311	1311	1311	1311
R-squared	0.34	0.18	0.17	0.34	0.16

Standard errors are in parentheses; *** *p* < 0.01, ** *p* < 0.05, * *p* < 0.1.

## Data Availability

Data will be made available upon reasonable request to the first author (cf2781@cumc.columbia.edu).

## Appendix A

**Table A1:** Distribution of answers for each item selected.

	Never	Sometimes	Often	Always
Do you enjoy the work?	25%	34%	21%	20%
Are you proud of your work?	34%	29%	20%	18%
Do you feel like you have the skills needed to do your job well?	13%	33%	31%	23%
Do you think others appreciate the work you do?	16%	45%	24%	15%
Do you feel under pressure to work faster and harder?	45%	35%	13%	7%
Do you get bored while at work?	40%	50%	7%	4%
Do you feel tired?	15%	58%	16%	10%
Do you feel that, if you wanted to, you could choose what to do and what not to do?	36%	42%	11%	11%
At work, do you feel that people watch over you to make sure you don’t get hurt?	38%	34%	15%	12%
Do people at work teach you what to do and how to do it?	18%	48%	19%	15%
Do you have free time each day to do just what you want?	30%	48%	12%	10%
Do you have lots of energy?	27%	41%	20%	11%
Do you have any difficulty sleeping?	80%	15%	3%	2%
Do you have trouble concentrating?	85%	12%	2%	1%
Do you feel sad and like crying?	53%	37%	7%	3%
Do you get very angry and often lose your temper?	47%	46%	5%	2%
Do you have little appetite or interest in food?	73%	22%	3%	2%
Do you feel tension in your body?	74%	21%	4%	1%
Do you feel dizzy?	46%	42%	9%	3%
Do you feel afraid or nervous?	60%	32%	5%	2%
Do you worry and think a lot?	41%	42%	11%	5%
Do you feel accepted by the other families around here?	25%	42%	24%	9%

## Appendix B

**Table A2:** Results of factor analysis: rotated factor loadings and uniqueness for the five-factor model.

Items	Factor 1 Emotionality	Factor 2 Self-Agency	Factor 3 Work-Related Self-Esteem	Factor 4Work-Related Stress	Factor 5 Supervision	Uniqueness
Do you enjoy the work?	0.00	−0.05	0.72	0.15	0.02	0.43
Are you proud of your work?	−0.06	−0.02	0.81	0.04	0.00	0.35
Do you feel like you have the skills needed to do your job well?	0.04	0.35	0.47	−0.05	−0.06	0.55
Do you think others appreciate the work you do?	0.04	0.31	0.32	−0.20	0.11	0.65
Do you feel under pressure to work faster and harder?	0.11	−0.06	0.02	0.51	−0.02	0.69
Do you get bored while at work?	0.23	−0.02	0.26	0.40	−0.03	0.59
Do you feel tired?	0.18	0.00	0.14	0.44	−0.02	0.66
At work, do you feel people watch over you to make sure you don’t get hurt?	0.04	0.05	−0.01	0.03	0.54	0.67
Do people at work teach you what to do and how to do it?	−0.03	−0.10	0.01	−0.03	0.84	0.36
Do you have any difficulty sleeping?	0.48	0.03	−0.03	−0.04	0.02	0.78
Do you have trouble concentrating?	0.47	0.02	−0.03	−0.09	−0.03	0.81
Do you feel sad and like crying?	0.52	−0.09	0.03	0.13	0.06	0.65
Do you get very angry and often lose your temper?	0.39	−0.12	0.01	0.01	0.05	0.83
Do you have little appetite or interest in food?	0.42	0.05	−0.07	−0.08	0.00	0.84
Do you feel tension in your body?	0.59	0.07	−0.02	0.03	−0.02	0.64
Do you feel dizzy?	0.52	0.00	0.00	0.01	−0.02	0.73
Do you feel afraid or nervous?	0.49	0.07	−0.03	0.05	−0.02	0.74
Do you worry and think a lot?	0.57	−0.01	−0.02	0.20	−0.02	0.57
Do you feel that, if you wanted to, you could choose what to do and what not to do?	−0.03	0.34	−0.07	0.25	0.01	0.84
Do you have free time each day to do just what you want?	0.04	0.41	−0.14	0.34	0.07	0.70
Do you have lots of energy?	0.01	0.67	0.00	−0.02	−0.11	0.61
Do you feel accepted by the other families around here?	−0.11	0.39	0.01	−0.03	0.07	0.81
**Share of total variance explained by each factor**	0.37	0.30	0.23	0.22	0.19	

**Table A3:** Factor correlation matrix.

	Factor 1 Emotionality	Factor 2 Self-Agency	Factor 3 Work-Related Self-Esteem	Factor 4 Work-Related Stress	Factor 5 Supervision at Work
**Factor 1 Emotionality**	1.00				
**Factor 2 Self-agency**	0.06	1.00			
**Factor 3 Work-related self-esteem**	0.18	0.56	1.00		
**Factor 4 Work-related stress**	0.52	0.17	0.38	1.00	
**Factor 5 Supervision at work**	0.15	0.54	0.36	0.08	1.00

## Appendix C. Linear Regressions Results, Detailed per Factor, and Block of Items

The following five tables display the results of the linear regressions at three levels, including only variables with *p*-values > 0.2 in the larger model in Table 2. Standard errors are in parentheses.

**Table A4:** Linear regression for emotionality factor.

	(1)	(2)	(3)
	Personal	Family	Work
Age	−0.059 ***	−0.045 **	−0.042 ***
	(0.018)	(0.018)	(0.016)
Gender	−0.116 **	−0.091 **	−0.18 ***
	(0.047)	(0.046)	(0.043)
Child has been sick over the past twelve months	−0.456 ***	−0.435 ***	−0.332***
	(0.049)	(0.047)	(0.043)
Family support index		0.057 **	0.052 **
		(0.025)	(0.022)
Child has fights with his household members		−0.287 ***	−0.114 **
		(0.049)	(0.045)
Child does not live with at least one of his parents		−0.197 ***	−0.191 ***
		(0.048)	(0.043)
Child has food or water at work			0.119 ***
			(0.045)
Child is engaged in hazardous work (ILO index)			−0.235 ***
			(0.023)
Child is a victim of violence at work: Work violence index			−0.216 ***
			(0.022)
Child’s main activity is casual work			−0.167 ***
			(0.064)
_cons	1.192 ***	1.226 ***	1.024 ***
	(0.267)	(0.263)	(0.237)
Observations	1311	1311	1311
R-squared	0.126	0.175	0.338

Standard errors are in parentheses; *** *p* < 0.01, ** *p* < 0.05.

**Table A5:** Linear regressions for self-agency factor.

	(1)	(2)	(3)
	Personal	Family	Work
Child is enrolled at school	−0.024	−0.131 **	−0.139 **
	(0.056)	(0.055)	(0.055)
Child has been sick over the past 12 months	−0.111 **	−0.112 **	−0.071
	(0.047)	(0.045)	(0.045)
Family support index		0.232 ***	0.225 ***
		(0.024)	(0.024)
Child has fights with household members		0.127 ***	0.156 ***
		(0.046)	(0.047)
Child does not live with at least one of their parents		0.112 **	0.107 **
		(0.045)	(0.045)
Child’s community views working children favorably		0.114 ***	0.127 ***
		(0.044)	(0.044)
Friend quality index		0.057 **	0.053 **
		(0.022)	(0.022)
Child is working alone			−0.12 ***
			(0.045)
Child’s monthly income (in USD)			0.178 **
			(0.082)
Child walks more than 30 min per day to work			−0.141 ***
			(0.05)
Child has food or water at work			0.056
			(0.047)
Child is engaged in hazardous work (ILO index)			−0.053 **
			(0.025)
Child is a victim of violence at work (index)			−0.061 ***
			(0.023)
Child’s main activity is casual work			−0.201 ***
			(0.066)
_cons	0.089	−0.013	−0.001
	(0.057)	(0.069)	(0.083)
Observations	1311	1311	1311
R-squared	0.066	0.147	0.18

Standard errors are in parentheses; *** *p* < 0.01, ** *p* < 0.05.

**Table A6:** Linear regressions for self-esteem factor.

	(1)	(2)	(3)
	Personal	Family	Work
Child is a girl	−0.052	−0.039	−0.146 ***
	(0.05)	(0.049)	(0.05)
Family support index		0.173 ***	0.145 ***
		(0.025)	(0.024)
Child’s community views working children favorably		0.082 *	0.104 **
		(0.049)	(0.048)
Child is working alone			−0.138 ***
			(0.049)
Child’s monthly income (in USD)			0.137
			(0.089)
Child walks more than 30 min per day to work			−0.281 ***
			(0.054)
Child has food or water at work			0.124 **
			(0.052)
Child is a victim of violence at work (index)			−0.148 ***
			(0.024)
Child’s main activity is casual work			−0.219 ***
			(0.075)
Child’s main activity is *khat* collection			−0.202 **
			(0.097)
Child’s main activity is domestic work			0.227 *
			(0.118)
_cons	0.024	−0.022	0.092
	(0.034)	(0.041)	(0.068)
Observations	1311	1311	1311
R-squared	0.055	0.093	0.163

Standard errors are in parentheses; *** *p* < 0.01, ** *p* < 0.05, * *p* < 0.1.

**Table A7:** Linear regressions for stress factor.

	(1)	(2)	(3)
	Personal	Family	Work
Age	−0.04 **	−0.036 **	−0.036 **
	(0.017)	(0.016)	(0.014)
Child is a girl	−0.056	−0.037	−0.126 ***
	(0.044)	(0.043)	(0.038)
Child is enrolled at school	0.162 ***	0.151 ***	0.088 *
	(0.055)	(0.054)	(0.047)
Family support index		0.05 **	0.047 **
		(0.023)	(0.02)
Child has fights with household members		−0.265 ***	−0.087 **
		(0.045)	(0.04)
Child’s community favorably view working children		−0.158 ***	−0.079 **
		(0.042)	(0.037)
Child’s monthly income (in USD)			−0.157 **
			(0.07)
Child walks more than 30 min per day to work			−0.187 ***
			(0.043)
Child has food or water at work			0.099 **
			(0.04)
Child is engaged in hazardous work (ILO index)			−0.173 ***
			(0.021)
Child is a victim of violence at work (index)			−0.255 ***
			(0.02)
_cons	0.485 *	0.636 **	0.616 ***
	(0.255)	(0.251)	(0.219)
Observations	1311	1311	1311
R-squared	0.077	0.125	0.342

Standard errors are in parentheses; *** *p* < 0.01, ** *p* < 0.05, * *p* < 0.1.

**Table A8:** Linear regressions for supervision factor.

	(1)	(2)	(3)
	Personal	Family	Work
Age	−0.041 **	−0.023	−0.023
	(0.018)	(0.018)	(0.018)
Child is enrolled at school	0.038	−0.066	−0.089
	(0.06)	(0.058)	(0.057)
Asset and wealth index		0.046 *	0.033
		(0.024)	(0.024)
Family support index		0.272 ***	0.26 ***
		(0.025)	(0.025)
Child has fights with household members		0.137 ***	0.117 **
		(0.048)	(0.048)
Child does not live with at least one of their parents		−0.074	−0.081 *
		(0.049)	(0.048)
Child’s community views working children favorably		0.07	0.064
		(0.046)	(0.045)
Child is working alone			−0.186 ***
			(0.047)
Child walks more than 30 min per day to work			−0.117 **
			(0.051)
Child has food or water at work			0.109 **
			(0.049)
Child’s main activity is casual work			−0.254 ***
			(0.069)
Child’s main activity is domestic work			0.074
			(0.11)
_cons	0.559 **	0.33	0.434
	(0.28)	(0.27)	(0.272)
Observations	1311	1311	1311
R-squared	0.032	0.124	0.153

Standard errors are in parentheses; *** *p* < 0.01, ** *p* < 0.05, * *p* < 0.1.
